# A PSMA-targeted theranostic approach is unlikely to be efficient in serous ovarian cancers

**DOI:** 10.1186/s13550-021-00756-z

**Published:** 2021-02-09

**Authors:** Nicolas Aide, Laurent Poulain, Nicolas Elie, Mélanie Briand, Florence Giffard, Cécile Blanc-Fournier, Florence Joly, Charline Lasnon

**Affiliations:** 1 Nuclear Medicine Department, University Hospital, Caen, France; 2 UNICAEN, INSERM 1086 ANTICIPE, Normandy University, Caen, France; 3 Comprehensive Cancer Centre F. Baclesse, Biological Ressources Centre OvaRESSOURCES, UNICANCER, Caen, France; 4 UNICAEN, SF 4206 ICORE, CMABIO3, Normandy University, 14000, Caen, France; 5 Department of Bio-Pathology, Comprehensive Cancer Centre F. Baclesse, UNICANCER, Caen, France; 6 Department of Uro-Gynecological Oncology, Comprehensive Cancer Centre F. Baclesse, UNICANCER, Caen, France; 7 Nuclear Medicine Department, Comprehensive Cancer Centre F. Baclesse, UNICANCER, Caen, France

**Keywords:** Epithelial ovarian carcinoma, Human FOLH1 protein, PSMA, Nuclear medicine

## Abstract

**Purpose:**

Until now, results evaluating the expression of PSMA in ovarian cancer were sparse and contradictory. The aim was to reinvestigate the feasibility of a PSMA targeted theranostic approach in epithelial ovarian cancers with data from the tumour bank of a referring cancer centre.

**Materials and methods:**

The OvaRessources Biological Resources Center database was screened from January 2004 to December 2017 to seek patients referred for the initial management of a serous epithelial ovarian cancer and for whom peritoneal histological samples were available in the tumour bank. Immunodetection of PSMA was performed to assess its cellular and neovascular expression. Slides were controlled by a certified pathologist, recorded as tiled tiff images and processed to compute the proportion of DAB stained surface.

**Results:**

Of the 51 patients identified by the database screening, 32 patients were included resulting in 57 samples (32 pre-chemotherapy and 25 post-chemotherapy histological samples). Nine patients were chemo-sensitive, 10 were partially chemo-sensitive and 13 were chemo-resistant/refractory. In the entire dataset, the expression of PSMA was quasi-inexistent: %DAB_PSMA_ = 0.04 (± 0.12) %. There was no significant difference in the %DAB_PSMA_ of sensitive, partially sensitive and resistant/refractory patients. There was also no significant difference in %DAB_PSMA_ in tumours before and after chemotherapy in the 25 patients for whom both samples were available.

**Conclusion:**

The present work demonstrates that PSMA expression is negligible and a fortiori non-sufficient to ensure its usefulness as a prognosticator or a target for a theranostic strategy in ovarian cancers.

## Introduction

Ovarian cancer is responsible for more than 150,000 deaths per year worldwide and is the leading cause of death from gynaecological cancer. One in 100 French women will develop ovarian cancer before the age of 75, and in 80% of cases the diagnosis is made at an advanced stage of abdominal dissemination [1–3]. The resistance of tumours to treatment with cisplatin-based chemotherapy protocols in ovarian cancer leads to a real problem of therapeutic strategy [4, 5]. First-line surgery and chemotherapy can achieve response rates close to 80%. However, among patients whose tumours were initially sensitive to treatment, 3/4 relapse within 18 months and develop drug resistance [6–8]. The introduction of new treatments and the evolution of protocols over the past thirty years have only slightly improved overall survival, which remains below 40% at 5 years. The identification of drug-resistant patients and the development of new therapeutic strategies capable of overcoming drug resistance are therefore two major challenges.

PSMA (prostate-specific membrane antigen) is a type II transmembrane glycoprotein which was initially shown to be expressed in prostate epithelial cells. This protein is overexpressed in almost all prostate cancers and is currently widely exploited for imaging and treatment of prostate cancer around the world. It has also been shown for a long time now that PSMA is not specific for prostate cancer and is expressed in the neovasculature of a large variety of solid cancers [9–11], thus being a potential target for the development of an antineovasculature treatment. Moreover, a recent review summed up the rational and current status on imaging of non-prostate cancers using PSMA-targeted radiotracers [12] and cited the work of Wernicke et al. [13] that demonstrated PSMA expression in primary ovarian tumours as well as in ovarian carcinoma metastases.

Therefore, the aim of the present work was to reinvestigate the expression of PSMA in epithelial ovarian cancers from the tumour bank of a referring cancer centre to evaluate the feasibility of a PSMA targeted theranostic approach in the future.

## Materials and methods

**Fig. 1:**
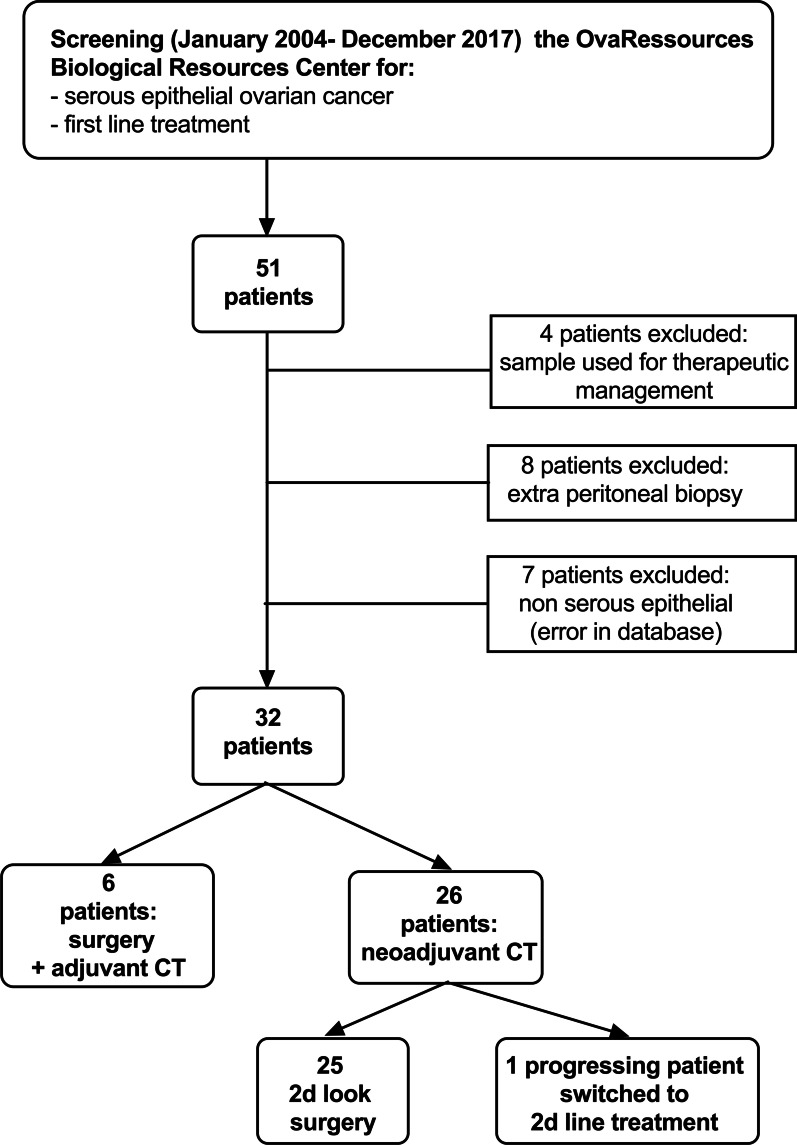
CONSORT diagram of the study population

The OvaRessources Biological Resources Center (NF-S 96900 quality management, AFNOR No. 2016: 72860.5) database was screened from January 2004 to December 2017 to seek patients referred to our centre of reference for the initial management of a serous epithelial ovarian cancer and for whom peritoneal histological samples were available in the tumour bank (Fig. 1). All first lines of treatment were considered. The biological collection was declared to the MESR (Ministry of Education, Health and Research, France, No. DC 2010-1243). We obtained written informed consent for patients still alive under the agreement of the ethical committee “North-West III” (CPP). For other patients (deceased or lost to the follow-up), an authorization was obtained from CPP to use their samples. Patients were classified in 3 groups according to their time of relapse after chemotherapy with cisplatin:

- Between 0 and 6 months (refractory and/or resistant groups),
- Between 6 and 12 months (partially sensitive)
- Beyond 12 months (sensitive).

Immunodetection of PSMA was performed to assess its cellular and neovascular expression. Immunohistochemistry was performed on paraffin embedded tumour tissues using a Ventana Discovery XT autostainer on 4 μm-thick sections. Slides were deparaffinised with EZPrep buffer at 75 °C for 8 min, and epitopes were unmasked at 95 °C for 8 min and 100 °C for 4 min in EDTA buffer. Sections were incubated 40 min at 37 °C with PSMA antibody (ab133579, Abcam, 1/1000). Secondary antibody (Omnimap Rabbit) was incubated for 16 min at 37 °C. After washes, staining was performed with 3,3′-diaminobenzidine (DAB), and sections were counterstained with hematoxylin. Whole slide images were digitized at 20 × (0,5 µm/pixel) using the ScanScope CS scanner (Leica Biosystems, Nussloch, Germany). The slides were controlled by a certified pathologist.

They were recorded as tiled tiff images. For each image, regions of interest (ROI) were drawn using the ImageScope software (Leica Biosystems) in order to select only tumour tissues and remove the artefacts. The images were processed to compute the proportion of DAB stained surface as follows:

$$\frac{Surface_{DAB}}{Surface_{Total}} \times 100$$

Quantitative data are presented as mean (± SD). A Kruskal–Wallis non-parametric test was used to compare PSMA expression between chemo-sensitive, partially chemo-sensitive, chemo-resistant and refractory tumours. A Wilcoxon test was used to compare the expression of PSMA before and after chemotherapy. Graph analysis and statistical analysis were performed on XLSTAT software (XLSTAT 2047: Data analysis and statistical solutions for Microsoft Excel. Addinsoft (2017)). For all statistical tests, a two-tailed p value of less than 0.05 was considered statistically significant.

## Results

The database screening allowed identifying a panel of 51 patients. Finally, 32 patients were included. Causes of exclusion can be seen in Fig. 1. Patients and tumour characteristics can be found in Table 1. All patients gave their consent for the use of their histological materials as well as their computerized medical data.

**Table 1:** Patients and tumours characteristics

N°	Age (y)	FIGO	Grade	Mutation	Treatment	PSMA %DAB 1	PSMA %DAB 2	Interval between samples (months)	Relapse	Time of relapse after CT (months)	Death	OS (months)
1	46	III	III	No	Surgery—ACT	0.703	–	–	Yes	0.3	No	19.2
2	66	III	III	BRCA1	NACT—cytoreductive surgery—ACT	0.005	0	3.3	Yes	18.0	Yes	70.8
3	83	III	III	No	NACT—exploratory surgery—ACT	0.109	0	3.0	Yes	7.6	No	64.7
4	58	IV	III	Not available	NACT—exploratory surgery—ACT	0.123	0	4.0	Yes	46.6	No	69.0
5	56	III	III	No	Surgery—ACT	0	–	–	Yes	32.0	No	84.7
6	62	III	III	No	NACT—cytoreductive surgery—ACT	0.009	0.003	2.9	Yes	7.9	No	61.0
7	63	III	III	Not available	NACT—cytoreductive surgery—ACT	0.017	0.081	3.7	Yes	4.7	Yes	37.5
8	61	III	II	Not available	Surgery—ACT	0.001	–	–	Yes	10.0	Yes	49.4
9	61	III	III	No	Surgery—ACT	0	–	–	Yes	1.0	Yes	17.8
10	61	III	III	Not available	NACT—Progression, second line of ttt	0.003	–	–	Yes	0.8	Yes	5.5
11	55	III	III	No	NACT—cytoreductive surgery—ACT	0.003	0	3.8	Yes	4.0	Yes	20.8
12	64	III	III	No	NACT—cytoreductive surgery—ACT	0	0.004	3.2	Yes	13.2	Yes	45.4
13	63	III	III	Not available	Surgery—ACT	0.028	–	–	Yes	1.1	Yes	8.5
14	73	III	III	No	NACT—cytoreductive surgery—ACT	0	0	4.1	Yes	4.8	No	53.9
15	48	III	III	No	NACT—exploratory surgery—ACT	0.008	0.033	4.1	Yes	3.0	Yes	16.6
16	74	IV	III	No	NACT—cytoreductive surgery—ACT	0.036	0	3.0	Yes	14.6	Yes	46.2
17	79	III	III	No	NACT—cytoreductive surgery—ACT	0.078	0.082	3.3	Yes	3.4	No	40.2
18	58	III	III	No	NACT—cytoreductive surgery—ACT	0.016	0.006	3.4	Yes	9.9	Yes	23.4
19	70	III	III	No	NACT—exploratory surgery—ACT	0.022	0.002	5.0	Yes	6.9	No	20.4
20	66	III	III	BRCA2	NACT—cytoreductive surgery—ACT	0.014	0.018	4.9	No	–	No	91.0
21	48	III	III	BRCA1	NACT—cytoreductive surgery—ACT	0.008	0	3.2	Yes	10.6	Yes	38.1
22	65	III	III	Not available	NACT—exploratory surgery—ACT	0.001	0	5.7	Yes	7.9	No	27.8
23	72	III	II	No	NACT—cytoreductive surgery—ACT	0	0.428	3.7	Yes	11.0	Yes	32.7
24	76	III	III	No	NACT—cytoreductive surgery—ACT	0.010	0.002	4.4	Yes	25.9	Yes	53.6
25	68	III	III	Not available	NACT—cytoreductive surgery—ACT	0.002	0.008	4.1	Yes	13.9	Yes	52.1
26	66	III	III	No	NACT—cytoreductive surgery—ACT	0.001	0.001	4.3	Yes	6.0	Yes	102.8
27	76	III	III	Not available	NACT—exploratory surgery—ACT	0.053	0.065	6.0	Yes	2.3	Yes	8.3
28	74	III	III	No	NACT—cytoreductive surgery—ACT	0	0.006	5.2	Yes	9.4	Yes	31.4
29	67	IV	III	Not available	NACT—cytoreductive surgery—ACT	0.016	0.554	5.3	Yes	3.4	No	27.9
30	74	III	III	No	NACT—cytoreductive surgery—ACT	0.019	0.049	4.7	Yes	18.2	No	47.7
31	82	III	III	No	NACT—cytoreductive surgery—ACT	0.027	0	4.4	Yes	8.6	Yes	33.3
32	83	III	III	Not available	Surgery—ACT	0.031	–	–	Yes	0.5	Yes	21.4

CT, chemotherapy; NACT, neoadjuvant chemotherapy; ACT, adjuvant chemotherapy; ttt, treatment

Six patients underwent cytoreductive surgery followed by platinum/taxane-based chemotherapy. Twenty-six patients had neoadjuvant chemotherapy (NACT). Interval cytoreductive surgery followed by adjuvant IV platinum/taxane-based chemotherapy ± bevacizumab was feasible in nineteen patients after 3 or 4 courses of NACT, except for 2 patients who had a total of 6 NACT courses. After NACT, 6 patients could not undergo complete interval surgery and therefore received maintenance platinum/taxane-based chemotherapy ± bevacizumab. However, for these 6 patients, interval histological samples were available from the exploratory surgery. Finally, one patient progressed through NACT and switched to pegylated liposomal doxorubicin hydrochloride. Post-NACT samples were available for all patients with the exception of the one who progressed through NACT.

Overall 57 samples were studied: 32 pre-chemotherapy and 25 post-NACT histological samples. The mean time-span between the initial and post-NACT histological samples was equal to 4.1 ± 0.8 months (range: 2.9–6.0). The mean time-span between NACT and surgery was equal to 35.6 ± 15.9 days (range: 24–63).

The mean follow-up time was equal to 3.5 ± 2.0 years. At time of the database completion, 31 patients (96.9%) had experienced relapse and 20 patients (62.5%) had died.

Among the 32 included patients, 9 were classified chemo-sensitive, 10 partially chemo-sensitive and 13 chemo-resistant or refractory. Focusing on the 25 patients with two available samples, there were 8 chemo-sensitive, 9 partially chemo-sensitive and 9 chemo-resistant or refractory tumours.

**Fig. 2:**
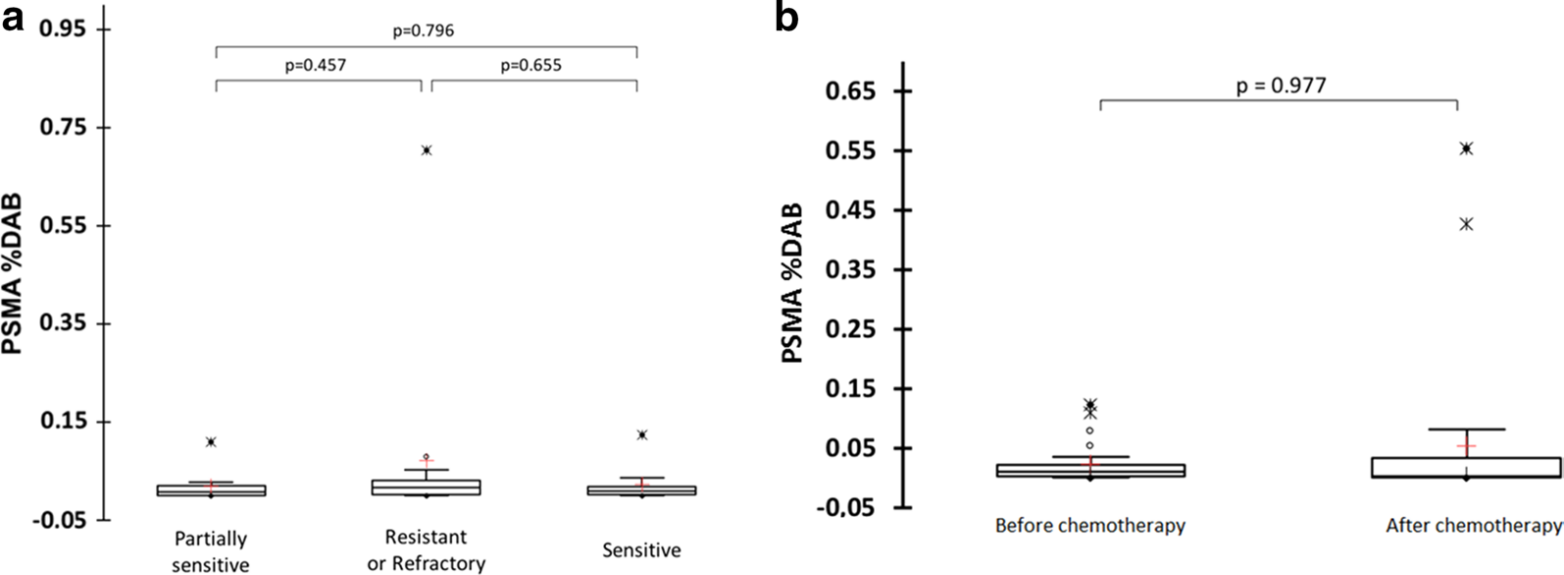
Comparison of PSMA expression represented as a proportion of DAB (**a**) between sensitive, partially sensitive and resistant/refractory ovarian tumours and (**b**) between pre-chemotherapy and post-chemotherapy samples. Lines denote median values, 10th and 90th percentiles, red crosses denote mean value

**Fig. 3:**
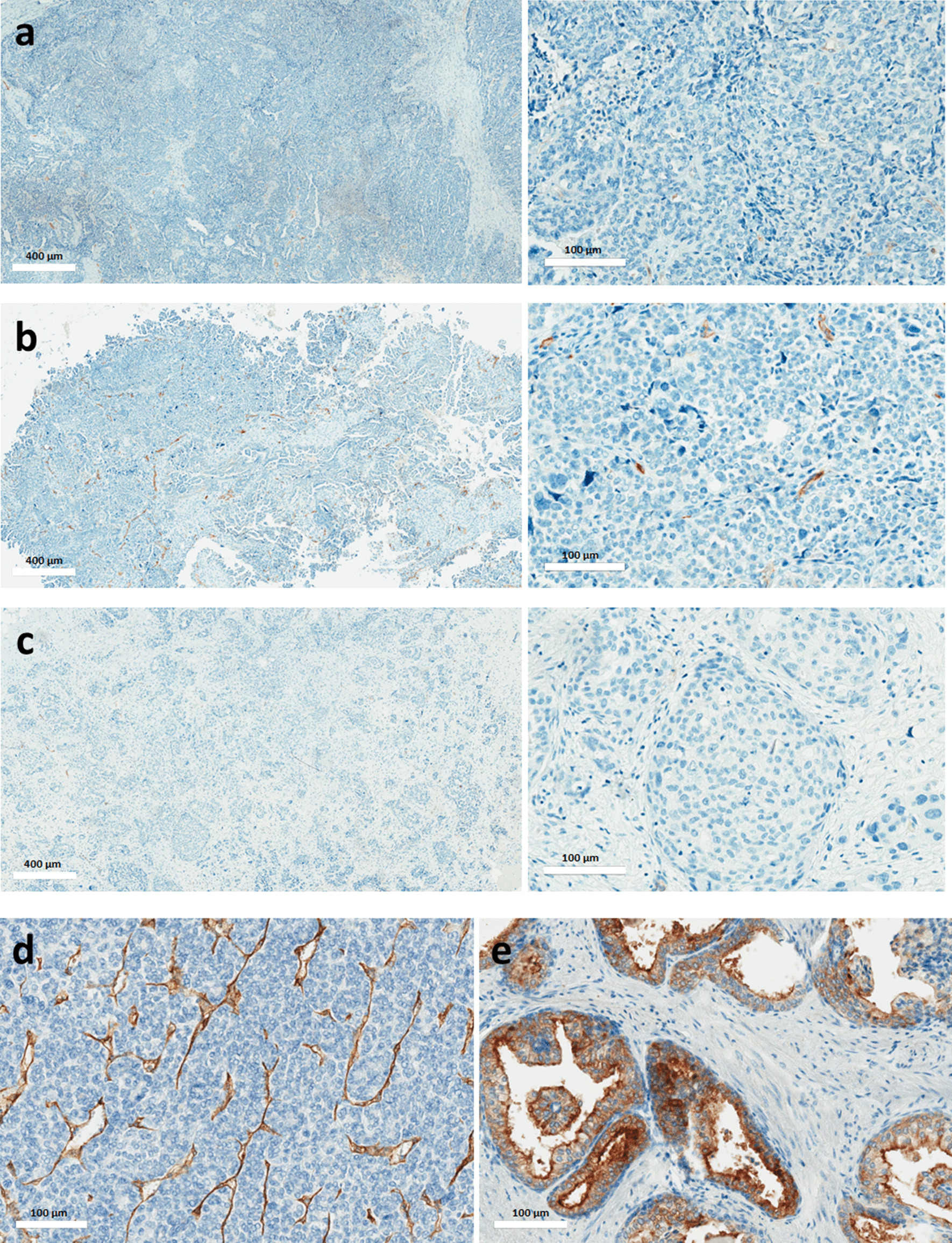
Representative slides of pre-chemotherapy PSMA immuno-staining in a sensitive (**a**), a partially sensitive (**b**) and a resistant (**c**) serous epithelial ovarian cancer. A very partial immuno-staining of tumour neovascularization can be seen in panel **b**. Lower panels correspond to positive controls: refractory thyroid cancer (**d**) and normal prostate tissue (**e**). For refractory thyroid cancer (**d**), we observe an immuno-staining of neovascularization, while for prostate cancer (**e**) there is an immuno-staining of prostate glandular cells

In the entire dataset, the expression of PSMA was quasi-inexistent with a mean PSMA %DAB equal to 0.04 (± 0.12) %. There was no significant difference in the PSMA %DAB of sensitive, partially sensitive and resistant/refractory patients as displayed in Fig. 2a (*p* = 0.751). A representative iconography of pre-chemotherapy immunochemistry slides for each group of patients is presented in Fig. 3. There was also no significant difference in PSMA %DAB in tumours before and after chemotherapy in the 25 patients for whom both samples were available (Fig. 2b). Our immunohistochemical protocol included positive controls that demonstrated strong PSMA expression in other normal (prostate) or tumour (thyroid) tissues, as exemplified on the Figs. 3d, e.

## Discussion

The two main findings of the present work are the quasi-absence of PSMA expression within serous epithelial ovarian cancers, whatever its degree of resistance to chemotherapy and the non-evolution of PSMA expression during the treatment course.

Contradictorily Wernicke et al. [13] described a PSMA expression in the neovasculature of primary ovarian tumours, 31% of tumours exhibiting an expression of more than 50% in tumour vasculature. Regarding the tumour cellular expression of PSMA, they found that nearly 50% of primary ovarian tumours they studied were positive. Similarly to our study, all were high grade serous carcinomas, in which 10 to 50% of the cells were positive with both cytoplasmic and membrane expression. We definitely did not find such results in our population, which is very wondering. Our immunohistochemical protocol cannot be incriminated since we detected strong PSMA expression in other normal (prostate) or tumour (thyroid) tissues, used as positive internal controls, as exemplified on the Fig. 3. Besides, looking carefully at figures from the paper of Wernicke AG et al., the chosen iconographies do not seem to fully support the point, visually showing weak cellular and neovascular expressions. Wernicke AG et al. also described more intense neovascular expression of PSMA in metastases than primary lesions. In contrast, the majority of them were negative at the cellular level. Even if we did not explore this specific point, it is worth noticing that we had a larger data bank from which to support our findings and that it was the first time that the evolution of PSMA staining during the course of treatment was explored.

Conversely, our results are concordant with Kinoshita Y. et al. demonstrating that ovary stromal cells stained strongly, whereas ovary carcinoma did not express PSMA [14]. However, this study included only 5 normal ovaries and 1 ovarian carcinoma tissue sample. Also, data from the human protein atlas, which is a Swedish-based program initiated in 2003 with the aim to map all the human proteins in cells, tissues and organs using integration of various omics technologies [15], seems to confirm that ovarian carcinomas are PSMA negative cancers (see: https://www.proteinatlas.org/ENSG00000086205-FOLH1/pathology/ovarian+cancer).

On the other hand, a preclinical study demonstrated that a low level of PSMA expression in non-prostatic tumours was sufficient for in vivo tumour targeting and imaging [16]. However, investigations were conducted on melanoma and non-small cell lung cancer that in this specific study displayed higher PSMA expression than ovarian cancer, questioning the SPECT/CT images and quantification that could have been obtained with ovarian carcinoma xenografts. It is worth noticing that the number of patients included in the present study is limited, but it comes from a centre of reference for management of ovarian cancers and it is for now the largest currently available database exploring PSMA expression in ovarian cancers. In view of these preliminary results which left us with little hope of conclusive results, we decided not to pursue our investigation with a PET clinical trial as it was originally planned. However, PSMA probes are currently being investigated in clinical trials now recruiting in North America and to be completed next year (NCT03857087, NCT03811899, NCT03302156). We are looking forward to seeing if they will confirm our findings.

## Conclusion

Until now, results evaluating the expression of PSMA in ovarian cancer were sparse. The present work, using samples from the tumour bank of a referring cancer centre, demonstrates that PSMA expression is negligible and a fortiori non-sufficient to ensure its usefulness as a prognosticator or a target for a theranostic strategy in ovarian cancers.

## Data Availability

The data supporting the conclusions of this article will be made available by the authors, upon reasonable request.

## Abbreviations

PSMA: Prostate specific membrane antigen
ROI: Region of interest
DAB: Diamino-3,3′benzidine tetrachlorhydrate
PET: Positron emission tomography
